# Demonstration of high value care to improve oral health of a remote Indigenous community in Australia

**DOI:** 10.1186/s12955-020-01300-8

**Published:** 2020-02-24

**Authors:** Sanjeewa Kularatna, Ratilal Lalloo, Jeroen Kroon, Santosh K. K. Tadakamadla, Paul A. Scuffham, Newell W. Johnson

**Affiliations:** 1 Australian Centre for Health Services Innovation, School of Public Health and Social Work, Queensland University of Technology, Brisbane, Australia; 2 School of Dentistry, The University of Queensland, Brisbane, Australia; 3 School of Dentistry and Oral Health, Giffith University, Gold Coast, Australia; 4 Menzies Health Institute Queensland, Griffith University, Gold Coast, Australia; 5 Faculty of Dentistry, Oral and Craniofacial Sciences, King’s College London, London, England

**Keywords:** Cost effectiveness, Oral health, Indigenous, Utility, Cost utility, QALY, Caries

## Abstract

**Background:**

The oral health of Indigenous children in remote communities is much worse than other population groups in Australia. Providing and maintaining an oral health service is challenging due to the remoteness of communities, the associated high cost, and the low retention of clinical staff. An annual preventive intervention delivered by fly-in clinicians may be a more cost-effective way to manage this problem. In this analysis we estimate the cost-effectiveness of an annual professional intervention for the prevention of dental caries in children of a remote Indigenous community in Far North Queensland.

**Methods:**

A cost-effectiveness analysis was conducted based on an annual preventive intervention protocol. This included treating all dental decay in those with disease, applying fissure sealants, a disinfectant swab, fluoride varnish and providing oral hygiene instructions and dietary advice to all participating school children. This study included an intervention group and a natural comparison group and both groups were followed-up for 2 years after the initial preventive intervention. A Markov model was built to assess the cost-effectiveness of the intervention compared with the usual care. Costs of treatment from the Queensland Department of Health were used and effectiveness was measured as quality-adjusted life years (QALYs) with the CHU-9D. One-way and probabilistic sensitivity analyses were conducted to identify key drivers and quantify uncertainty.

**Results:**

The preventive intervention was found to be highly cost-effective. The incremental cost per QALY gained was AU$3747. Probability of new caries and seeking treatment were identified as the main drivers of the model. In probabilistic sensitivity analysis intervention was cost effective in 100% of simulations.

**Conclusion:**

An annual preventive intervention for remote Indigenous communities in Australia is a highly cost-effective strategy to prevent dental caries and improve the quality of life of children.

## Introduction

The oral health of Aboriginal and Torres Strait Islander children (hereinafter respectfully referred to as Indigenous children) in Australia is in a dire state compared with other communities [1]. Similar to many other chronic disease conditions such as diabetes, heart failure, lung cancer and mental health issues, the effects of dental caries are more prevalent and severe within Indigenous communities [1]. Dental caries is a chronic disease process and frequently life-long. It is most commonly recorded by its effects: demineralisation of tooth substance progressing to cavitation and infection unless arrested by improved diet and oral hygiene, provision of preventive measures, or restoration of cavities [2]. On average remote Indigenous children (5–9 years) experienced 5 decayed, 1 missing and 1.3 filled deciduous tooth surfaces, with almost 60% having a dmfs> 0. In the permanent dentition (9–14 years), the average was 1.7 decayed, 0.1 missing and 0.7 filled tooth surfaces, with almost 59% having a DMFS> 0 [3]. Similar to other countries, the prevalence of caries lesions in Australia shows a consistent social pattern [4]. Low socio-economic factors and specific geographical areas have a strong association with high rates of dental caries, thereby increasing the plight of Indigenous children [3, 5–7]. Lack of water fluoridation is cited as part of the explanation for the high caries experience observed in children living in remote Indigenous communities [8].

It is important to find and consistently implement preventive interventions to reduce the activity and sequelae of the caries process in children and their subsequent adult life. Policy decisions by state governments to allow communities to decide on water fluoridation have not helped in this endeavour [8]. Many small communities in Queensland no longer use water fluoridation plants based on cost and cultural beliefs, which has led to increased caries, higher treatment costs, and a lower quality of life for children. Oral hygiene and dietary cautions are some of the primary preventive measures to reduce the activity of the caries process, however, these require effective behavioural interventions [9]. Attempts to discourage the intake of sugary foods and drinks have met with little success, this has led to increased caries experience in children in these, and many other communities worldwide. Active preventive strategies are important in this context to save teeth, improve quality of life, and reduce treatment costs [9].

The model of treatment provision depends on finite available resources. Many communities in Far North Queensland are served by fly-in fly-out oral health workers who spend only a few days in the community. The treatment provided is mainly reactive rather than proactive. Clinical emergencies are prioritised across all age groups and there are difficulties in carrying out comprehensive treatment plans. It is difficult to attract and retain qualified dental professionals to work full-time in remote communities. As such, a high staff turnover is common. However, these resources could be used more effectively in short, intensive, annual preventive interventions, as suggested in this study. The treatment of carious teeth (when present), application of fissure sealants, fluoride varnish, oral hygiene instructions and dietary advice could be delivered in one or a few contemporaneous visits to remote communities [10].

Any new strategy must be proven safe, effective and cost-effective for long-term sustainability. Health economic evaluation provides a means to assess the cost-effectiveness (value for money) of any new intervention; however, its use for oral health intervention is rare [11]. In this analysis we estimate the cost-effectiveness of an annual professional intervention for the prevention of childhood caries in a remote Indigenous community in Far North Queensland.

## Methods

The study was conducted in the Northern Peninsula Area (NPA) of Far North Queensland following the published protocol [10]. The study was undertaken with the formal permission of the Elders and Mayor of the NPA Council, and with the active participation of Queensland Health, Education Queensland, the local Community Health Service, School Heads and staff. Community residents were employed to liaise with families, and were particularly valuable in explaining and obtaining consents, and in transporting children between schools and the treatment facilities. A series of subsequent visits have been made to the community to present the results of the study and to cement ongoing relations.

This community had benefited from a period of public water fluoridation which ceased 4 years before the longitudinal preventive intervention described here [12]. All school children in the area were invited to take part. Of the approximately 600 children on school records, consent was obtained from the parents/guardians to participate in the study (*n* = 408). Of that, 196 children were consented to receive active treatment for existing carious lesions and subsequent preventive intervention. Although the study did not withhold treatment from any child, this provided an opportunity to have a natural comparison group to the group which received the preventive intervention. Children from school “Prep” to school year 12 (approximate ages 5 to 18 years) were included, examined and offered treatment of any cavitated carious lesions. A clinical team consisting of a dentist, and oral health therapist and two dental assistants was employed from the research budget. Education Queensland gave permission to examine children on school premises. Over 95% of the study sample was Indigenous. The baseline epidemiological survey and treatment phase was conducted in 2015 with 1- and 2-year follow-up visits in 2016 and 2017.

At baseline all consented children were examined and their caries status recorded using the International Caries Detection and Assessment System (ICDAS-II) [13]. Those with active carious lesions who consented were treated by the project clinical team who spent 3 months in the community. Treatment was conducted in either the dental clinic of the local hospital, or a school-based mobile dental clinic. All cavitated carious lesions present were treated, untreatable teeth extracted and indicated pits and fissures sealed where indicated. Following completion of the treatment plans povidone-iodine and fluoride varnish was applied, with oral hygiene and dietary instructions provided by the clinicians. This was called an annual “Big Bang” preventive intervention. During the 2016 and 2017 follow-up visits epidemiological surveys were conducted and the preventive intervention re-applied to the intervention group. Any child with new carious lesions was referred to local public oral health services for treatment.

The primary outcome of the intervention was the number of prevented caries lesions. This was measured as new tooth surfaces with a lesion. The identification process used ICDAS-II methodology which records sound surfaces as “0”, first visual change in enamel as “1”, distinct visual change in enamel as “2”, enamel breakdown as “3”, dentinal shadowing as “4”, a distinct cavity with visible dentine as “5” and an extensive cavity as “6”. ICDAS-II codes of 1–2 were regarded as incipient lesions; codes 3 to 6 were considered as surfaces with established carious lesions. A secondary outcome measure was the quality of life of participants with carious lesions. OHIP-14 [14], an oral health specific quality of life measure, and the CHU-9D, a generic paediatric multi-attribute utility instrument [15] were used to determine this. CHU-9D enables the calculation of utility values for health states, which can then be used to estimate the quality-adjusted life years (QALYs) based on duration in each health state. The CHU-9D scoring algorithm [16] using Australian population preferences, was used to calculate utility values for the caries health states. CHU-9D utility values and the number of caries prevented were used as the outcome measures in the economic evaluation.

### Model

**Fig. 1:**
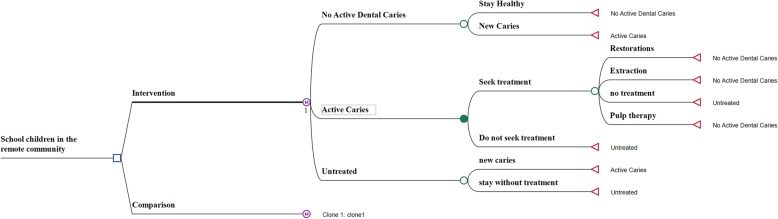
Markov model

A Markov model (Fig. 1) was built to analyse the cost-effectiveness of this annual preventive intervention strategy. This is a health state transition model with mutually exclusive health states. A health system perspective was used for the analysis. The model included 5- to 16-year-old children from this community, depicting their caries experience and cost of care. The model tracked the difference in caries experience between the intervention group and the usual-care comparison group (the group who did not receive the preventive intervention). The time horizon of the model was 10 years, commencing with children aged six which is the start of the mixed dentition stage and the first year of school. The model was completed at age 16 as this was the last reliable data, in terms of caries experience, that could be obtained from the school children of this community. Data of all children in the school was used including the students at “prep” class who are slightly below age six but above 5 years and considered them as six-year-old for the model.

The model had three health states: “No active dental caries”, “Active Caries” and “Untreated”, and started at a hypothetical time when the “Big Bang” intervention would be offered to all children. Children with caries in the comparison group would receive only the usual care provided by the health department. The “Big Bang” pathway was initiated in schools by proactively canvassing the children in their classrooms. With this preventive intervention the children should have less caries experience and acquire improved treatment-seeking behaviour. Children in the comparison group would not be exposed to these active preventive measures and would have to visit the local hospital clinic for treatment if in pain or otherwise motivated, for example by appearance.

At the start of the model, at age six, only a small percentage of the children were in the “No active dental caries” state. When a child developed caries, their health state changed to “New Active Caries” (ICDAS II codes 3–6). Model assumed Incipient caries lesions (ICDAS II codes 1 and 2) did not require any treatment as it was anticipated preventive intervention would reverse initial damage. For those that presented for treatment, general costs associated with a dental clinic were included in the model. These costs included clinical examinations, radiographs, plaque and calculus removal, oral hygiene instructions and dietary advice. We estimated all children would incur examination radiography costs, but only a portion would incur other costs. They would then undergo one of three treatment options: restoration, pulp therapy or extraction. Once treatment was completed their state returned to “No active dental caries”. Participants who could not receive treatment, or who did not seek treatment, were placed in the “Caries Untreated” state. They remained untreated or developed new caries lesions and moved to the “New Active Caries” state. All participants of the intervention group were offered fissure sealant (where indicated), povidone iodene, and fluoride varnish as the intervention. For the comparison group, these expenses were not incurred.

The model was validated using null and extreme values of important variables. Main probabilities of the model were used in two way sensitivity analysis with utility of “New Active Caries” health state and cost of restoration. The results of model validation are presented in Supplementary material.

### Transition probabilities

As dental caries is a slowly progressing disease we assumed that movement between “No active dental caries” and “New Active Caries” states would occur annually. We also assumed that the “Big Bang” intervention would be provided annually, during epidemiological surveys conducted in the schools, at which time children in need would be referred to the local hospital for treatment. These assumptions were based on the probability of some children developing carious lesions in the intervening period. The probability of children in the intervention and comparison groups developing new caries was used in the model to determine the movement between “No active dental caries” and “New Active Caries” states. Caries experience of different age groups from six to 16 years was assessed and decided to use the overall probability value for the base case analysis. The highest and the lowest probability of caries experience observed in both the intervention and comparison groups were used in the sensitivity analysis. The probability of seeking treatment was estimated from the experience of our longitudinal observations in the community and the National Child Oral Health Survey 2012–2014 [3]. We estimated that 90% of the intervention group and 68% of the comparison group would seek dental treatment [3]. The difference here is that the intervention is a proactive approach to identifying caries, with a clinician directly examining the mouth. In the usual care scenario the child or parent will identify the lesion after a cavity has formed (e.g. based on pain or gross appearance). For the sensitivity analysis, the 95% confidence interval reported in the survey was considered as the low and high values for the comparison group. For the intervention group, the low value was the base case value of the comparison group and the high value was 100% as there is a possibility all children would be examined and treated. However, the model does not incorporate the possible complicated and expensive treatment resulting from late diagnosis. All restoration types (one surface, two surface, three surfaces, and crowns) were considered as “restorations”. The rates calculated from the follow-up data were converted to probability using the rate-to-probability function in TreeAge (TreeAge Software Inc., Williamstown, Massachusetts, USA). The new caries experience rates observed in the study after the two-year follow-up visit were thus converted to an annual probability in the model.

### Costs

All costs are presented in Australian dollars as of 2018 (AU$1 ~ US$ 0.72 ~ Euro 0.63). The cost of care for the intervention group in “No active dental caries” state included examination, radiography, fissure sealant and disinfectant followed by varnish application. Costs of care for the comparison group at the “No active dental caries” state was zero due to them not being examined as in the intervention. Bitewing radiographs were taken as the costs for any radiography. All costs were taken from the fee schedule provided by the Queensland Health Department’s Office of the Chief Dental Officer.

The percentage of children who needed fissure sealant was recorded, as was the average number of teeth that needed treatment. A weighted mean cost of all restorations was included in the model. The restoration cost varied depending on the type of restoration (metallic or adhesive), tooth location (posterior or anterior) and the number of surfaces requiring restoration. Of the total number of restorations conducted, proportion of different types of restorations were calculated. The cost for each restoration was multiplied by relevant proportion before mean cost was estimated as a weighted mean. The lowest and highest costs of restorations were used in the sensitivity analysis. The mean cost of tooth extraction was used in the model. The lowest and highest cost of tooth removal in the fee schedule was used in the sensitivity analysis. Pulp therapy included direct pulp capping and pulpotomy. The weighted mean cost of pulp therapy was used for the base case analysis. The low cost of direct pulp capping and high cost of pulpotomy were used in the sensitivity analysis.

### Utility

The primary effectiveness measure of the model was quality-adjusted life years (QALYs). We used a utility value derived from CHU-9D as the outcome measure. The mean CHU-9D score of all children with ICDAS-II scores of 4 to 6 was 0.9. In a previous study this value was reported as 0.87 [17]. We used the value of 0.87 as the base case value and 0.9 and 0.8 [18] for the sensitivity analysis.

### Analysis

Using expected cost analysis, the mean cost per treated child over the 10-year period was calculated. For the base case analysis, the incremental cost-utility ratio (ICER) was calculated by dividing the incremental cost by incremental QALYs. The secondary outcome measure was number of avoided caries. The number of prevented caries and cost per prevented carious lesion were calculated. Results for a cohort of 500 children aged from six to 16 years was presented to reflect this. Using a baseline proportion of children with ICDAS-II codes of 3 to 6 that needed treatment, a 95% CI was calculated to estimate the low and high value for the probability of caries in both groups [19]. The low and high probability of treatments (restorations, extractions and pulp therapy) were estimated with ±15% of the base value. All costs and utilities were discounted at 5% per year as recommended by the Medical Services Advisory Committee’s Technical Guidelines, Australia [20].

Deterministic sensitivity analysis was performed to account for the uncertainties of parameter inputs into the model using low and high values of parameters. A tornado diagram was produced to illustrate the variables that most affected the results. A one-way sensitivity analysis was conducted using identified low and high values of the base case variables. As the base case was run with the comparison group starting with a higher proportion of caries, a sensitivity analysis was conducted making this similar to the intervention group. Another analysis was performed with none of the children having any caries at the start of the model (aged 6 years). This could reflect the natural caries experience, as permanent teeth begin to erupt at this age.

To quantify the results in relation to the uncertainty of the model inputs, probabilistic sensitivity analysis (PSA) was carried out, and randomly resampled 10,000 times from probability distributions for each parameter. Cost estimates used γ-distributions, and probabilities and utility weights used β-distributions. A triangular distribution was used for the probability of seeking treatment, as only three values were available. Only the important probabilities, cost and utilities were defined with distributions and used in the PSA.

## Results

There were 196 children in the intervention and 212 children in the comparison group. Most of the children were in the mixed dentition stage, aged between 6 to 12 years. In this sample less than 10% of children had no caries experience. ICDAS-II score of 1 to 2 were not considered as active caries in this analysis (Table 1).

**Table 1:** Demographic characteristics of the sample

Variable	Intervention (*N* = 196)	Comparison (*N* = 212)
	N (%)	N (%)
Gender^a^		
Males	87 (44.8)	98 (46.7)
Females	107 (55.3)	112 (53.3)
Age group		
< 6	39 (19.9)	42 (19.8)
6–12	128 (65.3)	139 (65.6)
> 12	29 (14.8)	31 (14.6)
ICDAS group^b^		
0	18 (9.2)	8 (3.8)
1–2	162 (82.7)	190 (89.6)
3–6	141 (71.9)	168 (79.2)
School^a^		
Primary school	148 (80.4)	171 (81.4)
High school	36 (19.6)	39 (18.6)

^a^Totals might not be equal to the actual number of participants due to missing values

^b^The column total and percentage does not equal the total number of participants, as some participants could have both ICDAS 1–2 and ICDAS 3–6 carious surfaces,

Model input values (Table 2), shows that the comparison group had a higher actual prevalence of caries compared with the intervention group, which we assumed would be the norm if a “Big Bang” preventive intervention is provided annually. The 2-year follow-up found that 63.7% of children had developed new caries (ICDAS_II 3 to 6) in the comparison group, compared to 47.9% in the intervention group. Children in the usual care scenario needed to travel to the hospital dental clinic which was open for one or 2 days a week. This would have a negative impact on their treatment-seeking behaviour. The National Oral Health Study indicated that only 68% of children would seek treatment [21]. However, in the intervention scenario where the prevention is provided within the school, with proactive encouragement of teachers, we assumed that 90% of children would seek treatment. This value was confirmed with our intervention group, where more than 90% of children consented to treatment. Of the total caries diagnosed, 40% received restorations, 6% received extractions, and 4% received pulp therapy. The highest total cost of the treatment was for restoration of teeth. The weighted mean of all restorations was applied as the base case value ($145). It was estimated that 50% of children would require plaque and calculus removal, and 25% would require additional oral hygiene and dietary advice.

**Table 2:** Model input values

Variable	Base case	Low value	High value	Distribution	Reference
Probabilities (P)					
P of Caries in comparison	0.80	0.74	0.85	Beta	Study data
P of Caries in intervention	0.71	0.65	0.78	Beta	Study data
P of new caries in comparison	63.7^a^	35.7^a^	71.4^a^	Beta	Study data
P of new caries in intervention	47.9^a^	31.6^a^	54.5^a^	Beta	Study data
P of seek treatment in comparison	0.68	0.63	0.73	Triangular	Ref
P of seek treatment in intervention	0.90	0.68	100	Triangular	Study data and ref
P of restorations	0.40	−15%	+ 15%	–	Study data
P of extractions	0.06	−15%	+ 15%	–	Study data
P of pulp therapy	0.04	−15%	+ 15%	–	Study data
Cost					
Cost of examination	$53	48	78	–	QH
Cost of restoration	$145	117	229	Gamma	QH
Cost extraction	$195	84	442	–	QH
Cost of pulp therapy	$77	31	538	–	QH
Cost of fissure sealant	$47	42	47	–	QH
Cost of radiography	$38	31	95	–	QH
Cost of varnish	$30	–	–	–	QH
Cost of plaque removal	$55	–	–	–	QH
Cost of calculus removal	$91	82	91	–	QH
Cost of oral hygiene instructions	$50	–	–		QH
Cost of dietary advice	$37	–	–	–	QH
Utility					
Utility of caries	0.87	0.8	0.9	–	Study data and [17]
Utility of health	1.00	0.95	1.0	–	

^a^observed probability after 2 year follow up period

The intervention was found to be cost-effective in the base case analysis. The incremental cost-effectiveness ratio was $3747 per QALY gained (Table 3). As this is well below the usual willingness-to-pay value per QALY of $50,000 [22] it can be concluded that this intervention was highly cost-effective. For a cohort of children (*n* = 500), the incremental cost of the intervention will be $333,000 in 10 years. However, the intervention will generate an additional 90 QALYs and prevent 180 caries lesions over 10 years. The net monetary benefit of the intervention ($461,529) was higher than the comparison group ($453,303), calculated using a value of $50,000 per QALY.

**Table 3:** Cost effectiveness results of 500 children living in Cape York over 10 years

Intervention	Costs	Incremental costs	QALYs	Caries lesions	Incremental QALYs	Incremental caries	ICER
Comparison	$175,500		4535	1010			
Intervention	$508,500	$333,000	4625	830	90	180	3747

### Markov cohort output

For the first cycle of the model, 29% of children in the intervention group were healthy. Due to the intervention over the 10 year period and continuous annual care (over 90% of children were offered treatment), 38.5% were completely healthy after 10 years. At the onset, 71% had active caries, which reduced to 17.6%. However, 44% were in the untreated stage at the end of 10 years. This is due to, in our model, 50% of children with ICDAS 3–6 carious lesions not being treated in each year. It is possible that treatment of the carious lesions was not predicted to be sought. In the comparison group, the healthy proportion increased slightly from 20 to 25.7%. There were 52.8% of children in the untreated category in that group.

**Fig. 2:**
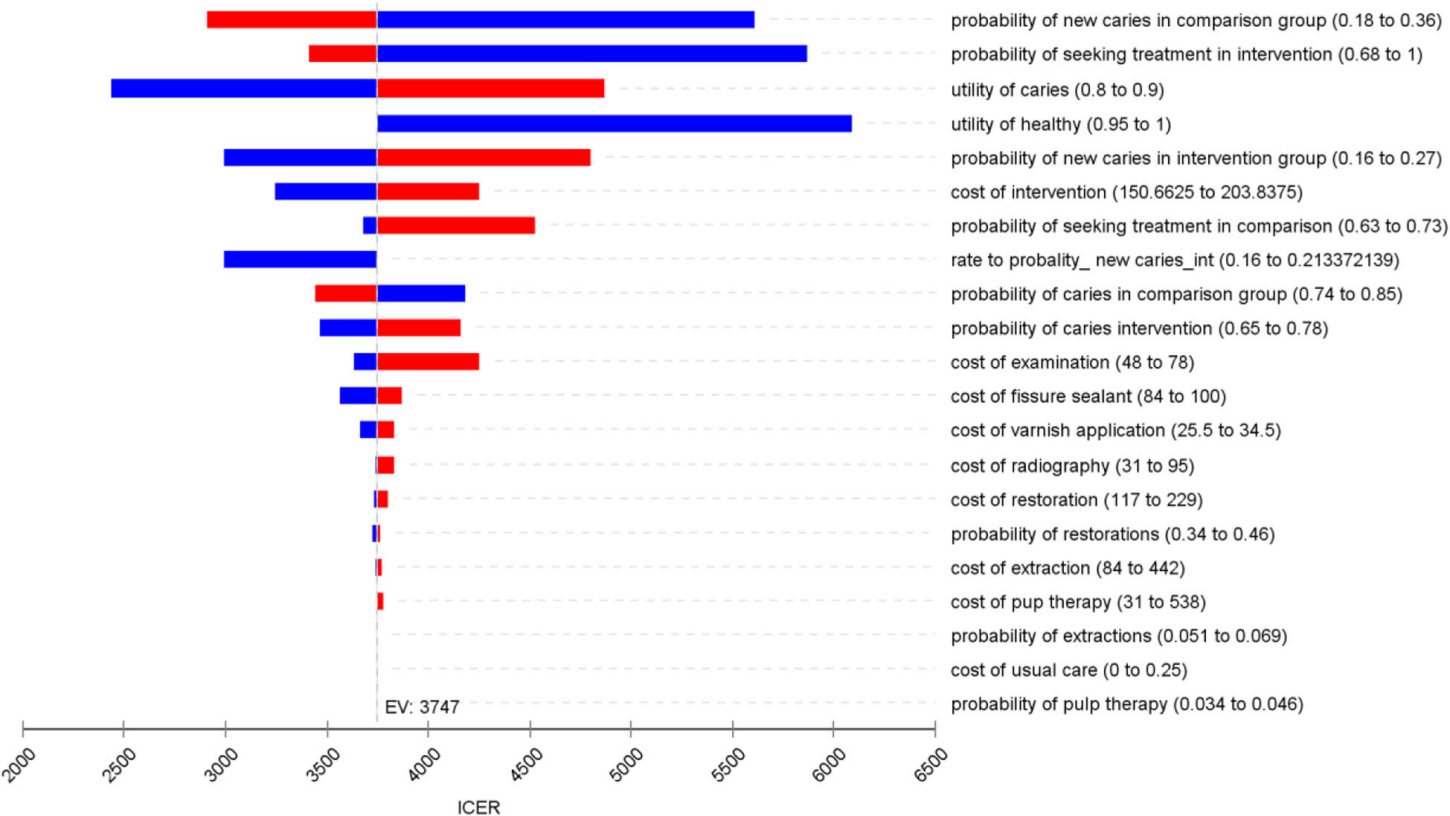
Tornado diagram showing results of one way sensitivity analysis

In all one way sensitivity analyses, the ICER remained positive for the intervention (Fig. 2). The key drivers of the model were the probability of new caries in the comparison group and the probability of seeking treatment in the intervention group. Increased utility values for the “Active Caries” state reduced the cost effectiveness of the intervention. The highest ICER reported was $5866 per QALY gained, when those seeking treatment in intervention group was set as the same probability to those in the comparison group (Table 4). When the probability of having caries in the intervention was made similar to comparison group (0.80), the ICER value was $4298 per QALY gained. When the probability of having caries was zero for the initiation of the model (all participants start with “No active dental caries”), the ICER was $5468. The change in probabilities for all factors did not make a substantial difference to the overall cost-effectiveness of the intervention (Table 4). This shows that the cost-effectiveness results for the intervention were relatively insensitive to changes in key assumptions and key parameters. The PSA results illustrate this finding (see next).

**Table 4:** One-way sensitivity analysis per 500 children in each group

Probabilities	Cost increment	QALY increment	Prevented caries	ICER
Initial caries in the comparison				
Low	337,000	80	185	4183
High	330,000	95	175	3441
Initial caries in the intervention				
Low	338,000	100	175	3461
High	327,500	80	185	4160
New caries in the comparison				
Low	366,500	65	120	5608
High	306,500	105	420	2910
New caries in the intervention				
Low	325,500	110	360	2993
High	342,500	70	85	4797
Seeking treatment in comparison				
Low	336,000	90	180	3682
High	308,500	70	180	4522
Seeking treatment in intervention				
Low	229,500	40	180	5866
High	380,500	110	180	3409
Cost				
Cost of restoration				
Low	331,500	90	180	3730
High	337,500	90	180	3798
Cost of fissure sealant				
Low	317,000	90	180	3562
High	344,000	90	180	3866
Utility				
Utility of caries				
Low	333,000	135	180	2435
High	333,000	70	180	4871

**Fig. 3:**
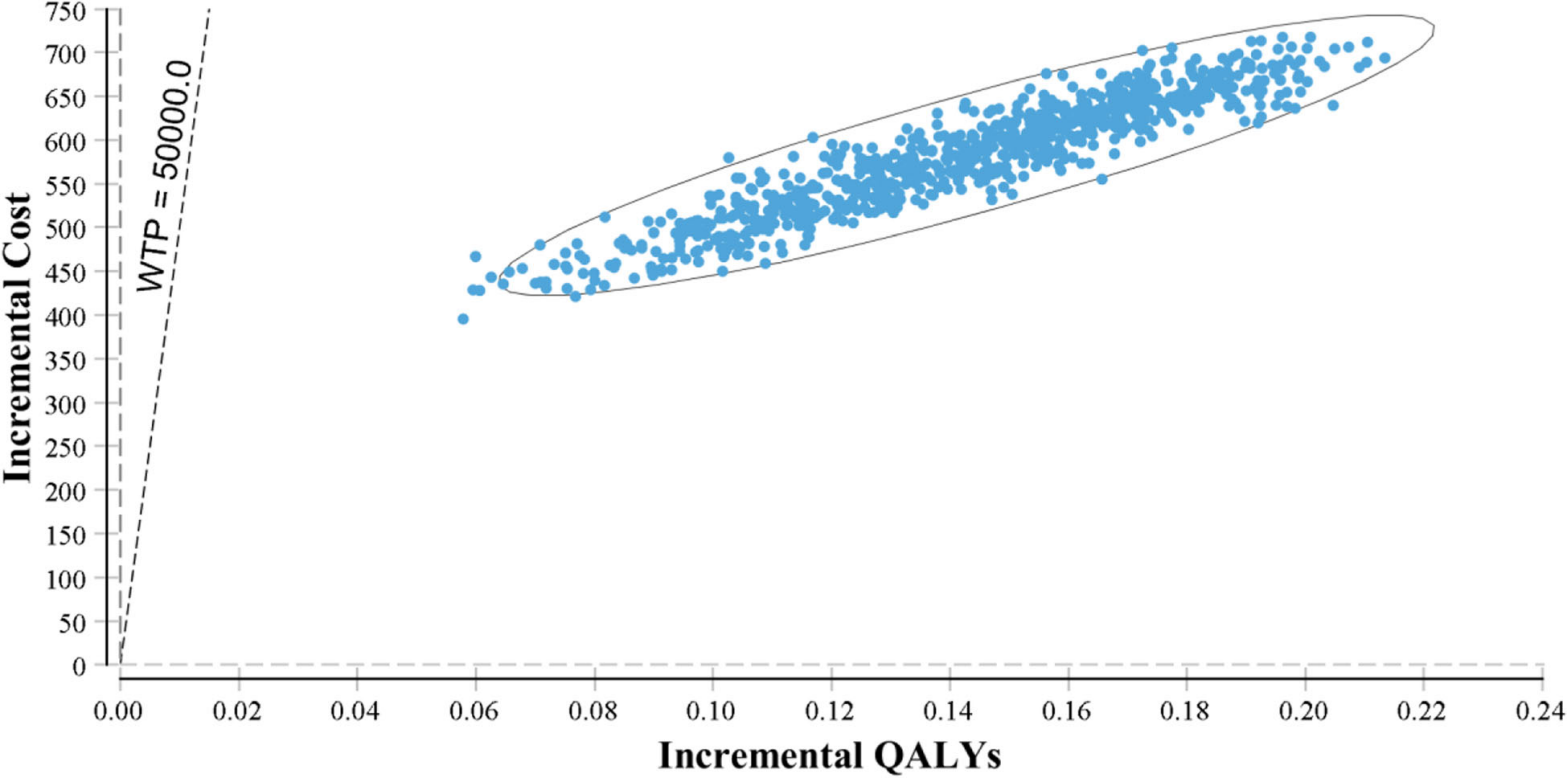
Probabilistic sensitivity analysis with 10,000 samples

The incremental cost-effectiveness scatter plot compares the incremental cost and incremental effectiveness (Fig. 3). The willingness-to-pay line (WTP) is shown for $50,000 [22]. All points appear to the right of the WTP line, which indicates the interventions were cost-effective in all iterations. PSA shows how the combined parameter uncertainty affects the overall confidence of the base case conclusions.

## Discussion

The annual “Big Bang” preventive intervention was found to be very good value for money compared with usual care in this remote Indigenous community. The new approach to preventive care would incur an additional cost but would substantially improve the quality of life of Indigenous children in remote areas. The pivotal factor was the effectiveness of fissure sealant and the proactive restoration of all dental decay.

From a practical perspective, could annual preventive care, as outlined in this paper, replace the routine care provided by dental clinics in community hospitals? We believe that annual interventions in schools should complement the existing care. Health departments could consider organising teams of mobile clinicians to travel to schools in remote areas, set up clinics in dental vans, screen all children, treat, fissure seal, apply varnish, and provide hygiene and dietary advice for all children. This would reduce the high caries rate and reinforce good oral hygiene and treatment-seeking behaviour in children and their parents. It may also reduce the workload of hospital dental clinics, which could provide planned preventive and maintenance care, instead of the current high workload caused by emergency management [23]. Remote communities such as the Northern Peninsula Area (NPA) might not need a full-time dentist in this scenario, and as such, the Health Department could more efficiently allocate existing resources to best manage the service needs.

Although more costly, the “Big Bang” intervention was more effective than the usual care received by the comparison group. The higher treatment consent observed in the intervention group is an important factor in this result. In a routine treatment scenario, Health Department consent processes will not be as rigorous as in a one-off research project. If the treatment-seeking behaviour of patients could be improved by motivated community workers and school staff, the number of carious lesions would decrease. The improvement in quality of life associated with improved oral health would be a substantial incentive for sustainable funding from the Health Department.

Quality of life in terms of oral health is different to general health. Discomfort in chewing food, pain, irritation when food is lodged between teeth, bleeding gums, halitosis and concerns of appearance resulting from discoloured and extracted teeth are major issues that affect quality of life. The effect of oral health on the quality of life of children is difficult to measure. With children, pain, ability to chew, obtaining enough nutrition, effect on school work, and social factors are major aspects that affect quality of life. These dimensions are included in the quality of life measure (OHIP14) used in this study. However, for the economic evaluation, we also needed to use CHU-9D to measure the utility of oral health states experienced by children. CHU-9D is a validated generic multi-attribute utility instrument for children 7 years and older; however, its validity in measuring oral health specific utility is a contentious issue [17, 24]. Using a Western-type quality-of-life instrument to assess Indigenous populations may not capture the correct information [25]. Also, there are valid arguments as to whether CHU-9D is capable of capturing quality-of-life issues in this unique culture [26].

Currently, there are few economic evaluations of oral disease prevention. A recent review found there has been an increased the reporting of such studies since 2011 [11]. Cost utility analysis (CUA), which is the focus of the current analysis, uses QALYs to measure outcomes. To date, only 15 previous studies have used this methodology to conduct economic evaluations of oral health interventions [11]. Our study results indicate that modest funding could substantially improve the quality of life of a community. Mostly, cost-effectiveness studies in oral health have reported cost minimisation analysis instead of cost utility analysis [27]. The results for this community have contributed to policy recommendations from the Australian Medical Association in their recent publication (ref).

### Limitations

This cost-effectiveness analysis used probabilities of one intervention. This study was not a randomised clinical trial, and as such, associated biases are not controlled. Both groups could have had other clinical oral health exposure which could have impacted the effectiveness used in this evaluation. The costs and utilities, however, were accurately measured or derived from other published literature. The comparison group had a higher proportion of caries at the outset. Nevertheless, these factors did not have a significant effect on the results, and a subsequent sensitivity analysis using similar caries experience for both groups did not change the overall results.

## Conclusion

A single annual professional oral health strategy consisting of treatment and application of fissure sealants, povidone iodene and fluoride varnish is a very cost-effective approach to improve oral health in remote Indigenous communities.

## Supplementary information

**Additional file 1.** Model validation: Two way sensitivity analysis with null and extreme values.

## Data Availability

NA- Access to raw data is restricted for ethical reasons but applications may be made in writing to the Chief Investigator, who can seek permission from a relevant ethics committee.

## Abbreviations

CHU-9D: Child Health Utility nine Dimensions
CUA: Cost utility analysis
ICDAS-II: International Caries Detection and Assessment System II
ICER: Incremental Cost Effectiveness Ratio
NPA: Northern Peninsula Area
OHIP-14: Oral Health Impact Profile 14
PSA: Probabilistic Sensitivity Analysis
QALYs: Quality Adjusted Life Years
WTP: Willingness-To-Pay
