# Supplementary material for: Demonstration of high value care to improve oral health of a remote Indigenous community in Australia
Source: Health Qual Life Outcomes. 2020 Feb 24;18:43. doi: 10.1186/s12955-020-01300-8 (PMC7041200; doi:10.1186/s12955-020-01300-8)
Supplement: Supplementary file 1 — Additional file 1. Model validation: Two way sensitivity analysis with null and extreme values. [file 12955_2020_1300_MOESM1_ESM.docx]

Model validation: Two way sensitivity analysis with null and extreme values

| Variable 1 | Variable 2 |  | Cost | Incremental cost | effectiveness | Incremental Effectiveness | ICER | NMB | Dominance |
| --- | --- | --- | --- | --- | --- | --- | --- | --- | --- |
|  |  |  |  |  |  |  |  |  |  |
| P of Caries in comparison | Caries utility |  |  |  |  |  |  |  |  |
| Null (0) | (0.5) | Comparison | 250 |  | 7.92 |  |  | 364435 |  |
|  |  | Intervention | 1017 | 767 | 7.12 | -0.17 | -439 | 354931 | Dominated |
| Extreme (0.85) | (0.9) | Comparison | 351 |  | 9.28 |  |  | 463455 |  |
|  |  | Intervention | 1017 | 660 | 9.42 | 0.15 | 4473 | 470172 |  |
| P of Caries in intervention | Caries utility |  |  |  |  |  |  |  |  |
| Null (0) | (0.5) | Comparison | 351 |  | 6.43 |  |  | 321396 |  |
|  |  | Intervention | 1132 | 781 | 7.91 | 1.48 | 528 | 394570 |  |
| Extreme (0.78) | (0.9) | Comparison | 351 |  | 9.29 |  |  | 463998 |  |
|  |  | Intervention | 1006 | 655 | 9.41 | 0.12 | 5408 | 469400 |  |
| P of new caries in comparison | Caries utility |  |  |  |  |  |  |  |  |
| Null(0) | 0.5 | Comparison | 127 |  | 7.22 |  |  | 360673 |  |
|  |  | Intervention | 1017 | 890 | 7.12 | -0.10 | -9174 | 354931 | Dominated |
| Extreme (0.36) | 0.9 | Comparison | 405 |  | 9.26 |  |  | 462690 |  |
|  |  | Intervention | 1017 | 613 | 9.42 | 0.16 | 3784 | 470172 |  |
| P of new caries in intervention | Caries utility |  |  |  |  |  |  |  |  |
| Null (0) | 0.5 | Comparison | 351 |  | 6.43 |  |  | 321396 |  |
|  |  | Intervention | 986 | 635 | 7.97 | 1.53 | 414 | 397396 |  |
| Extreme (0.27) | 0.9 | Comparison | 351 |  | 9.29 |  |  | 463998 |  |
|  |  | Intervention | 1036 | 685 | 9.40 | 0.11 | 6236 | 468804 |  |
| P of seek treatment in comparison | Caries utility |  |  |  |  |  |  |  |  |
| Null (0) | 0.5 | Comparison | 0 |  | 5.30 |  |  | 265116 |  |
|  |  | Intervention | 1017 | 1017 | 7.12 | 1.82 | 560 | 354931 |  |
| Extreme (0.73) | 0.9 | Comparison | 400 |  | 9.32 |  |  | 465542 |  |
|  |  | Intervention | 1017 | 617 | 9.42 | 0.10 | 5879 | 470172 |  |
| P of seek treatment intervention | Caries utility |  |  |  |  |  |  |  |  |
| Null (0) | 0.5 | Intervention | 169 |  | 5.55 |  |  | 274430 |  |
|  |  | Comparison | 351 | 182 | 6.43 | 0.88 | 205 | 321396 |  |
| Extreme (1) | 0.9 | Comparison | 351 |  | 9.29 |  |  | 463998 |  |
|  |  | Intervention | 1112 | 761 | 9.46 | 0.17 | 4431 | 471819 |  |
| P of caries in comparison | Cost of restoration |  |  |  |  |  |  |  |  |
| Null (0) | 0 | Comparison | 192 |  | 9.30 |  |  | 464627 |  |
|  |  | Intervention | 920 | 728 | 9.25 | -0.05 | -1602 | 461627 | Dominated |
| Extreme (0.85) | 229 | Comparison | 406 |  | 9.06 |  |  | 452551 |  |
|  |  | Intervention | 1074 | 668 | 9.25 | 0.19 | 3484 | 461473 |  |
| P caries in intervention | Cost of restoration |  |  |  |  |  |  |  |  |
| Null (0) | 0 | Comparison | 269 |  | 9.07 |  |  | 453385 |  |
|  |  | Intervention | 1065 | 796 | 9.46 | 0.38 | 2070 | 471817 |  |
| Extreme (0.78) | 229 | Comparison | 398 |  | 9.07 |  |  | 453256 |  |
|  |  | Intervention | 1064 | 666 | 9.23 | 0.16 | 4229 | 460463 |  |
